# TRAIL receptor I (*DR4*) polymorphisms C626G and A683C are associated with an increased risk for hepatocellular carcinoma (HCC) in HCV-infected patients

**DOI:** 10.1186/1471-2407-12-85

**Published:** 2012-03-08

**Authors:** Christian Körner, Katarina Riesner, Benjamin Krämer, Marianne Eisenhardt, Andreas Glässner, Franziska Wolter, Thomas Berg, Tobias Müller, Tilman Sauerbruch, Jacob Nattermann, Ulrich Spengler, Hans Dieter Nischalke

**Affiliations:** 1 Department of Internal Medicine I, University of Bonn, Sigmund-Freud-Str. 25, 53127 Bonn, Germany; 2 Department of Gastroenterology, University Hospital Leipzig, Philipp-Rosenthal-Strasse 27, 04103 Leipzig, Germany; 3 Medical Clinic for Hepatology and Gastroenterology, Medical University Charité, Augustenburger Platz 1, 13353 Berlin, Germany

**Keywords:** TRAIL receptor I, DR4, Apoptosis, Polymorphism, C626G (rs20575), A683C (rs20576), HCV, HCC, Cancer

## Abstract

**Background:**

Tumour surveillance via induction of TRAIL-mediated apoptosis is a key mechanism, how the immune system prevents malignancy. To determine if gene variants in the TRAIL receptor I (*DR4*) gene affect the risk of hepatitis C virus (HCV)-induced liver cancer (HCC), we analysed *DR4 *mutations C626G (rs20575) and A683C (rs20576) in HCV-infected patients with and without HCC.

**Methods:**

Frequencies of *DR4 *gene polymorphisms were determined by LightSNiP assays in 159 and 234 HCV-infected patients with HCC and without HCC, respectively. 359 healthy controls served as reference population.

**Results:**

Distribution of C626G and A683C genotypes were not significantly different between healthy controls and HCV-positive patients without HCC. *DR4 *variants 626C and 683A occurred at increased frequencies in patients with HCC. The risk of HCC was linked to carriage of the 626C allele and the homozygous 683AA genotype, and the simultaneous presence of the two risk variants was confirmed as independent HCC risk factor by Cox regression analysis (Odds ratio 1.975, 95% CI 1.205-3.236; p = 0.007). Furthermore HCV viral loads were significantly increased in patients who simultaneously carried both genetic risk factors (2.69 ± 0.36 × 10^6^ IU/ml vs. 1.81 ± 0.23 × 10^6^ IU/ml, p = 0.049).

**Conclusions:**

The increased prevalence of patients with a 626C allele and the homozygous 683AA genotype in HCV-infected patients with HCC suggests that these genetic variants are a risk factor for HCC in chronic hepatitis C.

## Background

Nearly 3% of the world population, app. 180 million people, suffer from hepatitis C virus infection (HCV) thus representing a global health problem [1]. In most of the cases exposure to HCV results in chronic viral persistence. Progression of chronic hepatitis C leads to liver fibrosis and cirrhosis and is associated with an increased risk to develop hepatocellular carcinoma (HCC) [2,3]. HCC has become the third leading cause of cancer-related death worldwide [4-6], and in Western Countries chronic hepatitis accounts for the majority of HCCs. HCV proteins can interact with tumour suppressor proteins as well as with proteins involved in cell-cycle control, and these interactions may promote the development of abnormal cells in the liver.

Tumour development is normally prevented by the immune system, which eliminates transformed cells via induction of apoptosis by tumour necrosis factor-related apoptosis-inducing ligand (TRAIL) [7,8]. Binding of TRAIL to its cognate death receptors *DR4* and DR5 triggers activation of the apoptotic cascade, formation of apoptotic bodies and eventually to depletion of the apoptotic cell. *DR4* and DR5 are members of the tumour necrosis factor super family and are characterized by the existence of an extra-cellular cysteine-rich binding domain as well as an intra-cellular death domain essential for the transmission of the apoptotic stimulus.

Genetic alterations in death receptors might compromise apoptotic cell signalling and therefore contribute to the development of tumour cells. Several studies suggested an increased risk for cancer associated with single nucleotide polymorphisms (SNPs) in the *DR4 *gene [9-13]. However, the potential influence of *DR4 *gene mutations on the development of HCC has not been investigated so far. Therefore, we analysed the effect of the *DR4 *polymorphisms C626G (Thr209Arg, rs20575) and A638C (Glu228Ala, rs20576) on the occurrence of HCC in patients chronically infected with HCV.

## Methods

### Design and study population

A total of 393 patients with chronic hepatitis C virus genotype 1 infection from the Bonn and Berlin University Departments of Gastroenterology were enrolled into this study including 159 patients with HCV-associated HCC and 234 patients without HCC. In addition 359 HCV-negative healthy individuals and 56 patients with HCC caused by chronic hepatitis B served as control groups. All subjects in this study were of Caucasian descent. Further clinical and demographic characteristics are listed in Table 1.

**Table 1:** Patient characteristics and distribution of* DR4* genotypes

	Healthy controls	HCV(+) all without HCC	HCV(+) Cirrhosis (-) Without HCC	HCV(+) Cirrhosis (+) without HCC	HCV(+) Cirrhosis (+) with HCC	HBV(+) With HCC
**Numbers**	359	234	159	75	159	56
**Median age (range)**	59 (35-69)	52 (39-63)	46 (39-57)	55 (49-63)	62 (54-70)	60(24-83)
**Male Gender (%)**	51.4	66.2	65.2	69.7	58.6	75.0
**HCV infection (%)**	-	100	100	100	100	0
**HCV genotype 1 (%)**	-	100	100	100	100	-
**HCV viral load(10^6^ IU/ml) ^a)^**	-	2.04 ± 0.25	2.10 ± 0.29	1.86 ± 0.48	1.88 ± 0.36	-
**HBV infection (%)**	-	3.2	2.8	4.0	4.4	100
**BMI > 30 (%)**	-	17.1	15.0	21.7	26.2	21.4
**Diabetes (%)**	-	7.7	0.8	23.0	12.0	3.6
**rs20575 (C626G) ^b) ^**	349 (100)	233 (100)	159 (100)	74 (100)	159 (100)	56 (100)
**CC**	62 (17.8)	56 (24.0)	38 (23.9)	18 (24.3)	40 (25.2)	9 (16.1)
**CG**	166 (47.6)	108 (46.4)	74 (46.5)	34 (45.9)	87 (54.7)	30 (53.6)
**GG**	121 (34.7)	69 (29.6)	47 (29.6)	22 (29.7)	32 (20.1)	17 (30.4)
**Deviation from Hardy-Weinberg equilibrium ^c) ^**	*p* = 0.74	*p* = 0.29	*p* = 0.43	*p* = 0.49	*p* = 0.27	*p* = 0.59
**rs20576 (A683C) ^b) ^**	359 (100)	234 (100)	159 (100)	75 (100)	159 (100)	56 (100)
**CC**	19 (5.3)	10 (4.3)	6 (3.8)	4 (5.3)	5 (3.1)	1 (1.8)
**AC**	125 (34.8)	85 (36.3)	59 (35.0)	26 (34.7)	33 (21.9)	17 (30.4)
**AA**	215 (59.9)	139 (59.4)	94 (59.1)	45 (60.0)	121 (75.8)	38 (67.9)
**Deviation from Hardy-Weinberg equilibrium ^c) ^**	*p* = 0.88	*p* = 0.58	*p* = 0.49	*p* = 1.0	*p* = 0.17	*p* = 1.0

^a) ^Mean ± SEM, ^b) ^Number of patients (number/% of total), ^c) ^Fisher's exact test

Cirrhosis was diagnosed either by liver biopsy, transient elastography (stiffness > 15 kPa), and signs of portal hypertension (splenomegaly, esophageal varices, ascites). The diagnosis of HCC was made by contrast enhanced magnetic resonance imaging and computed tomography according to recently established diagnostic criteria [EASL 2009 and AASLD 2010 guidelines].

Informed consent was obtained from all patients. The study conformed to the ethical guidelines of the Helsinki declaration and had been approved by the University of Bonn ethics committee (reference number: 019/07).

### Diagnosis of HCV Infection

HCV antibodies were detected with a micro particle enzyme immunoassay (Axsym; Abbott) and confirmed by dot immunoassay (Matrix; Abbott). HCV RNA was detected with a nucleic acid purification kit (QIAamp Viral Kit; Qiagen, Hilden, Germany), followed by reverse transcription and nested polymerase chain reaction. Quantitative determination of HCV loads was done by branched DNA technology (Chiron, Emeryville, CA). HCV genotype was determined by the Innolipa II line probe assay (Innogenetics, Zwijndrecht, Belgium).

### *DR4 *Genotyping

Genomic DNA was extracted from 200 μl EDTA-blood using the QIAamp Blood Mini Kit (Qiagen, Hilden, Germany) according to the manufacturer's protocol. Determination of the *DR4 *gene polymorphisms C626G (rs20575) and A683C (rs20576) were performed by LightCycler real time PCR (Roche, Mannheim, Germany) using commercial LightSNiP (SimpleProbe) assays from TIB-MolBiol (Berlin, Germany) according to the manufacturer's recommendations.

### Statistical analysis

Genotype frequencies were determined and tested for consistency with Hardy-Weinberg equilibrium using an exact test. Allele and genotype frequencies were compared between cases and controls by Pearson's goodness-of-fit (χ^2^) test and Armitage's trend test, respectively (http://ihg2.helmholtz-muenchen.de/cgi-bin/hw/hwa1.pl).

Statistical analysis was performed with SPSS 18.0 (SPSS, Munich, Germany). Data are given as means ± SD, unless stated otherwise. Differences between groups were analyzed by t-test and Mann-Whitney-U test as appropriate.

To take into account potentially confounding risk factors of cirrhosis (age, gender, HBV infection, alcohol and obesity), univariate comparisons (ANOVA and chi2-statistics) followed by forward conditional logistic regression were performed. Parameters with univariate effects at *p* < 0.1 were entered into the multivariate analysis with *p* < 0.05 for inclusion and *p* > 0.1 for exclusion as selection criterion for parameters in the final statistical model.

## Results

**Figure 1:**
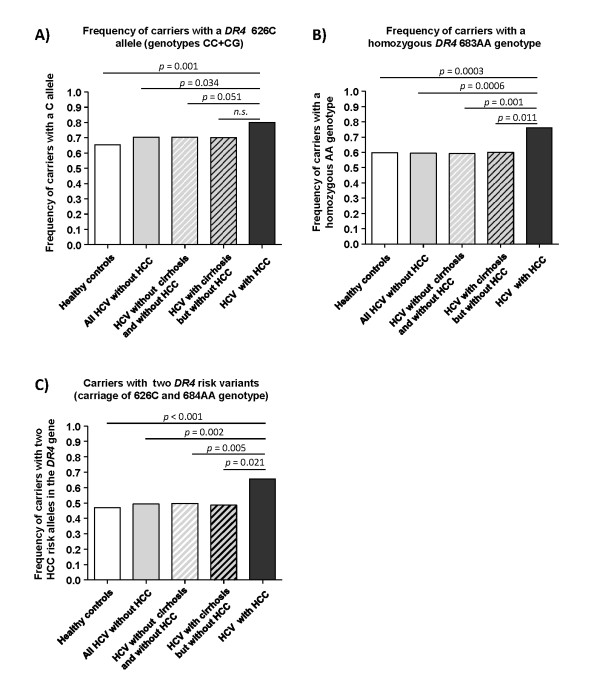
**Frequencies of *DR*4 risk alleles in HCV-infected patients with and without HCC**. Figure A shows the frequency of carriers with a *DR4 *626 C risk allele, and figure B the frequency of patients with the homozygous *DR4 *683AA genotype. Figure C shows the frequency of patients who carried either the 626CG or 626CC risk genotype in combination with the homozygous *DR4 *683AA genotype. Differences between the various patient groups were analyzed by chi^2 ^statistics.

The distribution of *DR4* C626 > G and A683 > C genetic variants matched the Hardy-Weinberg equilibrium in all analysed groups and subgroups. HCV-infected patients without HCC revealed a slightly higher allele frequency of the *DR4* 626C allele (47.2%) - corresponding to an increased prevalence of 626C carriers (70.4%) - than our healthy controls (allele frequency 41.5%; frequency of 626C carriers 65.3%; Figure 1A). These differences within the HCV-infected patient groups were apparently independent from the presence of cirrhosis and did not reach statistically significance.

Prevalence of carriers with a 626C allele (genotypes 626CG and CC) (79.9%) and 626C allele frequency (52.5%) were significantly increased in the patients with HCV-associated HCC (Figure 1A). The 626C allele frequency in patients with HCV-associated HCC was significantly different only from that in healthy controls (OR = 1.556, 95%CI: 1.192-2.031, *p *= 0.001).

Importantly, the increased prevalence of carriers with a 626C allele reached statistical significance both with respect to healthy controls (OR = 2.106, 95% CI: 1.348-3.290, *p *= 0.001) and to HCV-infected patients without HCC (OR = 1.670, 95% CI: 1.034-2.696, *p *= 0.034) (Figure 1A).

The distribution of A683 > C variants did not differ between healthy controls and HCV-infected patients without HCC irrespective from the presence of cirrhosis (Table 1). Patients with HCV-associated HCC had the highest frequency of the 623A allele (86.5%), which was significantly increased as compared to healthy controls (A allele frequency 77.3%, OR = 1.856 95% CI: 1.291-2.667, *p *= 0.0006) and HCV-infected patients without HCC (A allele frequency 77.6%, OR = 1.828, 95% CI: 1.243-2.686, *p *= 0.002). This deviation was strong enough to achieve statistical significance even when HCV-infected subgroups with and without cirrhosis were compared separately (cirrhosis: 77.3% A allele frequency, OR = 1.852, 95% CI: 1.126-3.045, *p *= 0.013; without cirrhosis: 77.7% A allele frequency, OR = 1.816, 95% CI: 1.201-2.746, *p *= 0.004).

**Figure 2:**
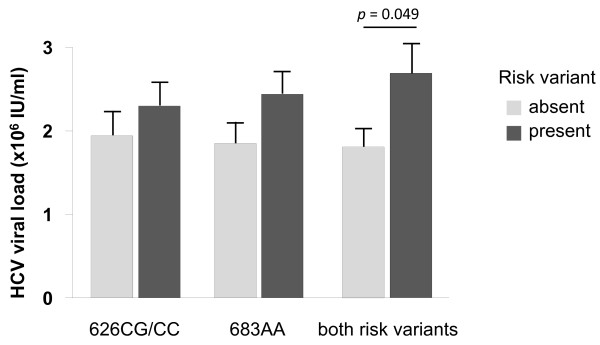
**HCV viral loads in patients with and without genetic *DR4 *risk variants**. This Figure shows HCV viral loads (Means ± SEM) in patients who carry the *DR4 *626CG or 626CC risk variant (left columns), the *DR4 *683AA risk variant (middle columns) and both *DR4 *risk variants simultaneously (right columns). Dark columns refer to patients with the studied risk factor, grey columns to those without the risk factor, respectively. Differences were compared by ANOVA.

These changes were due to a significant increase in the proportion of patients with the homozygous 683AA genotype in patients with HCV associated HCC (76.1%) as compared to healthy controls (59.9%, OR = 0.477, 95% CI: 0.314-0.725, *p *= 0.0003) and HCV-infected patients without HCC (59.4%, OR = 0.468, 95% CI: 0.300-0.730, *p *= 0.0006), irrespective whether cirrhosis was present (60.0%, OR = 0.480, 95% CI: 0.267-0.862, *p *= 0.011) or not (59.1%, OR = 0.462, 95% CI: 0.286-0.747, *p *= 0.001) (Figure 1B). Interestingly, HCV viral loads tended to be increased in patients who carried the genetic risk variants associated with polymorphisms C626G (2.44 ± 0.28 × 10^6 ^IU/ml vs. 1.85 ± 0.30 × 10^6 ^IU/ml, n.s.) and A683C (2.30 ± 0.27 × 10^6 ^IU/ml vs. 1.94 ± 0.26 × 10^6 ^IU/ml, n.s.), and this effect reached statistical significance in patients who simultaneously carried both genetic risk factors (2.69 ± 0.36 × 10^6 ^IU/ml vs. 1.81 ± 0.23 × 10^6 ^IU/ml, p = 0.049) (Figure 2).

Next, we checked whether variants in the TRAIL receptor I (*DR4*) gene were confirmed as independent HCC risk factors in HCV-infected patients when known risk factors such as age, sex, obesity, diabetes, HCV viral load and HBV co-infection were also taken into account (Table 2). Univariate analysis identified age (OR = 1.076 per year, 95% CI: 1.053-1.098, p < 0.0001), carriage of a 626C allele (OR = 1.670, 95% CI: 1.034-2.696, *p *= 0.035), the homozygous 683AA genotype (OR = 2.176, 95% CI: 1.390-3.407, *p *= 0.001) and the simultaneous presence of both genetic variants (OR = 1.940, 95% CI: 1.280-2.940, *p *= 0.002) as risk factors of HCC (Table 2). In the multivariate regression analysis age was found to be an independent risk factor for the development of HCC in chronic hepatitis C (OR = 1.074, 95% CI: 1.052-1.097, *p *≤ 0.001). More importantly, we could confirm that the simultaneous presence of a 626C allele in combination with the homozygous 683AA genotype was significantly associated with the occurrence of HCC in this continuative analysis (OR = 1.975, 95% CI: 1.205-3.236, *p *= 0.007) and could therefore identify the combined carriage of a *DR4* risk variant as new independent predictor for the development of HCC in chronic hepatitis C.

**Table 2:** Regression analysis for risk factors of HCC among patients with hepatitis C genotype 1

	Univariate analysis			
			95% Confidence interval	
Parameter	*p*-value	Odds ratio	Lower	Upper
Age ^§^	0.000	1.076	1.053	1.098
Diabetes mellitus	0.369	1.412	0.691	2.886
Gender (male)	0.135	1.430	0.915	2.236
HBV infection	0.626	1.295	0.460	3.646
HCV viral load (10^6^ IU/ml)	0.339	0.960	0.883	1.044
Obesity (BMI > 30)	0.153	1.668	0.867	3.211
C626G genotype ([CC+CG] vs. GG)	0.035	1.670	1.034	2.696
A683C genotype (AA vs. [AC+CC])	0.001	2.176	1.390	3.407
Combined 626C and 683AA risk genotype vs. all other combinations	0.002	1.940	1.280	2.940
	Multivariate analysis*			
			95% Confidence interval	
Parameter	p-value	Odds ratio	Lower	Upper
Age ^§^	0.000	1.074	1.052	1.097
Combined 626C and 683AA risk genotype	0.007	1.975	1.205	3.236

* including all significant parameters from univariate analysis, ^§ ^OR (95% CI) per year

To check, if our findings were specific for hepatitis C, we performed a similar analysis in our group of HCC patients, who had chronic hepatitis B. However, unlike hepatitis C-associated HCC the distributions of genetic *DR4* variants in HBV-associated HCC, healthy controls and HCV-positive patients without HCC were not significantly different (Table 1). In particular, the simultaneous combination of a 626C allele with the 683AA genotype was observed in rather similar frequencies in the groups with HBV-associated HCC (50.0%), hepatitis C without HCC (49.4%) and healthy controls (47.0%) while the difference in frequency between HBV-and HCV-associated HCC (65.4%) was significant (OR = 1.891, 95% CI: 1.020-3.506, *p* = 0.042).

## Discussion

Emerging evidence suggest an important role of TRAIL for control and elimination of HCV infection. TRAIL has been implicated in the death of HCV-infected but not normal liver cells [14]. In HCV infection its expression on natural killer cells is up-regulated by interferon, and is inversely correlated to HCV-RNA serum levels [15,16]. TRAIL can also induce cell death in hepatic tumour cells but expression of TRAIL receptors on human hepatocellular carcinoma is variable and frequently down-regulated [17]. Nevertheless, several studies suggest TRAIL receptor-mediated apoptosis to play a role in the elimination of tumour cells in human hepatocellular carcinoma [18,19].

In this study we found that the TRAIL receptor I wild type with threonine at amino acid position 209 (626C) and alanine at position 228 (638A) is associated with an increased risk of HCC in patients with chronic hepatitis C. Of note, it was particularly the combination of the risk genotypes 626CC or 626CG with the homozygous 683AA genotype that was identified as a new independent predictor for HCC. Interestingly, this association of *DR4* genetic variants and the risk to develop HCC was evident only for patients with chronic hepatitis C but not hepatitis B, suggesting a critical role of the aetiology underlying HCC. Both polymorphisms in the *DR4 *gene have already been reported to affect the risk for other types of malignancy. However, in a previous study investigating the role of the *DR4 *A683C polymorphism in chronic lymphocytic leukaemia, mantle cell lymphoma, prostate cancer, head and neck squamous cell carcinoma and bladder cancer an increased frequency of the CC genotype was apparently linked to malignancy [20]. In contrast, Frank *et al. *observed an increased percentage of carriers with the AA genotype in patients with breast cancer [10]. Moreover, they found an increased percentage of carriers of the 626CC genotype similar to our results. At present, it remains unclear which mechanisms underlie *DR4 *polymorphisms C626G and A683C to affect the risk for malignancy.

However, both amino acid exchanges are in the extracellular cysteine-rich domain of *DR4*. Therefore both genetic variants may lead to alterations in the TRAIL-binding domain and thus alter *DR4* affinity for TRAIL. Since TRAIL signalling presumably contributes importantly to the control of HCV infection, our finding of significantly increased HCV viral loads in carriers of both *DR4 *risk factors suggests that TRAIL-*DR4* signalling is less efficient in these patients. In line with this reasoning less efficient signalling of cell death in transformed cells and reduced susceptibility of transformed hepatocytes towards TRAIL-induced apoptosis would also facilitate HCC development. On the other hand, we cannot exclude the possibility that the increased HCC risk in HCV-infected patients carrying the C626G and A683C risk variants of TRAIL receptor I simply reflects less efficient immune control over HCV infection via TRAIL-mediated mechanisms. TRAIL is likely to affect tumour surveillance of transformed HCV-infected cells by the immune system and viral loads, once HCV infection has occurred [14]. On the other hand our data suggest that neither of the *DR4* polymorphisms affects susceptibility to HCV infection. This constellation can be explained by the fact that *DR4* expression is rather low on normal liver cells while up-regulated *DR4* expression following HCV infection sensitizes liver cells towards TRAIL-mediated apoptosis [14,21]. Thus, sufficient *DR4* expression on transformed liver cells appears to be a pivotal prerequisite for efficient tumour surveillance by the immune system, in line with recent clinical findings [17].

Therefore the role of TRAIL and *DR4 *polymorphisms should be further studied in patients whose risk for HCC is attributed to different aetiologies.

## Conclusion

Here, we provide first evidence that HCV genotype 1 infected carriers of a *DR4* 626C allele in combination with the *DR4* 683AA genotype have an increased risk for HCC indicating that TRAIL and its receptors contribute importantly to control of HCV infection and tumour surveillance by the immune system. The combined presence of the two *DR4* risk variants can help to identify HCV-infected patients with an increased risk for liver cancer, who may need more intensive cancer monitoring.

## Competing interests

The authors declare that they have no competing interests.

## Authors' contributions

KR and FW established and performed the real-time PCRs for *DR4* genotyping. BK, ME, TM and TB collected and analyzed clinical data and collected samples from the study population. HDN, US, and JN designed the study and did statistical analysis. CK and TS wrote the manuscript. All authors read and approved the final manuscript.

## Pre-publication history

The pre-publication history for this paper can be accessed here:

http://www.biomedcentral.com/1471-2407/12/85/prepub
